# The Role of Passion in Psychological and Cardiovascular Responses: Extending the Field of Passion and Positive Psychology in New Directions

**DOI:** 10.3389/fpsyg.2021.744629

**Published:** 2022-01-13

**Authors:** Robert J. Vallerand, Virginie Paquette, Christine Richard

**Affiliations:** Laboratoire de Recherche sur le Comportement Social, Université du Québec à Montréal, Montréal, QC, Canada

**Keywords:** passion, challenge and threat appraisals, physiological responses, Biopac, cardiovascular reactivity

## Abstract

The present study fills a void in research on passion by examining for the first time the role of passion in physiological responses. The aim of the study was to investigate the role of passion, and the mediating role of cognitive appraisals, in the psychological and physiological responses to a stressful situation related to one’s passion. Students (43 women, 12 men, *M* age = 27.21 years), who were passionate for their studies, completed the Passion Scale for their studies and the Cognitive Appraisal Scale (assessing perceptions of challenge/threat). Then, they engaged in an education task under stressful conditions, and a subsequent unrelated leisure task under no-stress. Physiological reactivity was measured throughout the entire session and their perceptions of situational vitality and positive and negative emotions were assessed directly after the education task. Results showed that harmonious passion (HP) positively predicted challenge appraisals that, in turn, were positively related to positive emotions, vitality, and positive cardiovascular adaptation while engaging in the stressful education task, but less so with the leisure task (unrelated to one’s passion for academia). On the other hand, obsessive passion (OP) positively predicted threat appraisals. In turn, threat appraisals were positively related to negative emotions, negatively associated with vitality, and not related to cardiovascular reactivity. The present findings suggest that HP creates the onset of an adaptive psychological and physiological response whereas the response is less adaptive with OP.

## Introduction

The last 20 years or so has seen an explosion of research in positive psychology (Seligman and Csikszentmihalyi, 2000), or the study of the factors that make life more fulfilling. One such factor is the concept of passion. Passion is defined as a strong inclination toward an activity that people like (or even love), find important, in which they invest time and energy, and that is part of identity (Vallerand, 2015). Indeed passion can fuel motivation, enhance wellbeing, and provide meaning in everyday life. However, passion can also arouse negative emotions, lead to inflexible persistence, and interfere with achieving a balanced, successful life. This is the duality of passion (Vallerand, 2015).

Vallerand et al. (2003) opened up the scientific field of passion when they published their article on the Dualistic model of passion (DMP) and a series of studies supporting it. Since then, hundreds of studies have been conducted and have supported the DMP. This model posits the existence of two types of passion: harmonious and obsessive. Harmonious passion (HP) originates from an autonomous internalization of the activity in identity and leads people to choose to engage in the activity that they love. It mainly leads to adaptive processes such as flow (i.e., a complete absorption of oneself in what one’s doing; Csikszentmihalyi, 1988), mastery goals (i.e., personal improvement goals; Elliot and Church, 1997), and task coping (i.e., problem-focused strategies altering a situation; Lazarus and Folkman, 1984). It also leads to adaptive outcomes such as positive emotions, subjective vitality, and wellbeing (for reviews, see Curran et al., 2015; Vallerand, 2015; Vallerand and Houlfort, 2019; Pollack et al., 2020). Conversely, obsessive passion (OP) originates from a controlled internalization in identity and leads people to experience an uncontrollable urge to engage in the activity that they love. It predicts less adaptive processes (Vallerand, 2015) such as avoidance goals (i.e., avoiding being worse than others; Elliot and Church, 1997), avoidance coping (i.e., behavioral and psychological withdrawal from a situation; Kowalski and Crocker, 2001), ego-involvement (e.g., self-esteem as contingent on one’s performance; Mageau et al., 2011), and less adaptive outcomes such as negative emotions and lower wellbeing (Vallerand, 2015).

Of particular interest for this study, research reveals that HP is positively related to challenge appraisals, while OP is positively associated with threat appraisals (e.g., Schellenberg et al., 2013; Schellenberg and Bailis, 2016; Lavoie et al., 2021). Challenge and threat appraisals are considered as two different constructs on two different continua (i.e., low to high challenge and low to high threat; Seery, 2013). According to the Transactional Model of Stress (Lazarus and Folkman, 1984), challenge appraisals occur when perceived coping resources match or exceed the perceived situational demands (Seery, 2011). The situation is then seen as an opportunity for gain or growth (Lazarus and Folkman, 1987). Challenge appraisals are positively related to positive emotions (e.g., Skinner and Brewer, 2002; Lavoie et al., 2021), vitality (i.e., feeling energized; Ryan and Frederick, 1997; Brown et al., 2017), and adaptive physiological responses (Tomaka et al., 1993) such as increases in cardiac output (CO; i.e., volume of blood in liters expelled from the heart per minute; Frings et al., 2012) and decreases in total peripheral resistance (TPR; i.e., the resistance to blood flow from all the circulatory system; Larkin and Cavanagh, 2016). On the other hand, threat appraisals occur when evaluated demands *exceed* one’s perceived coping resources (Seery, 2011). The individual then anticipates losses, and little or no gain (Lazarus and Folkman, 1987). Threat appraisals are positively related to negative emotions (e.g., Skinner and Brewer, 2002; Lavoie et al., 2021), negatively related to vitality (Brown et al., 2017), and they are associated with little change or minor decreases in CO, and little change or small increases in TPR (Tomaka et al., 1993). It is to be noted that task engagement no matter the type of cognitive appraisals, is associated with increases from baseline in heart rate (HR; i.e., the number of heart beats per minute; Seery, 2011).

Research on passion, just like the field of positive psychology, has generally focused on self-report measures. Although passion research has also used objective measures of performance and informant reports of other people’s wellbeing (e.g., Vallerand et al., 2007, 2008; Carbonneau and Vallerand, 2016), no research so far has looked at the role of passion in physiological responses. Looking at such responses would represent an important new direction for passion research that would bolster current findings and open up a window into new unchartered territories such as cardiovascular health. Research in positive psychology has recently started to look at physiological measures. For instance, research has started to look at the link between positive psychology variables such as gratitude (Enko et al., 2021) and emotions (Kaczmarek et al., 2021) and cardiovascular responses. Such efforts in positive psychology are important. However, the above studies did not include passion. Such research must be pursued in order to further extend the field of passion and positive psychology.

In line with the above, the goal of the present study was to extend past research on the role of HP and OP in self-reports of challenge/threat appraisals, positive and negative emotions, and subjective wellbeing by also assessing physiological measures such as cardiovascular reactivity (Blascovich, 2013). Further, we assessed these physiological measures both while participants engaged in a task related to their passion for academia (an education task) and in a control task (a novel leisure task). In line with past research (e.g., Lavoie et al., 2021) and the DMP (Vallerand, 2010, 2015), an integrated model was tested in which it was hypothesized that HP should foster adaptive cognitive responses of challenge appraisals, while OP should be positively related to cognitive threat appraisals. In turn, challenge appraisals should positively predict positive emotions and subjective wellbeing. Furthermore, in line with previous research (Tomaka et al., 1993), only challenge appraisals should predict challenge (adaptive) cardiovascular responses while engaging in the education task (task related to participants’ passion for academia), but less so with the leisure task (task unrelated to participants’ passion for academia). On the other hand, threat appraisals were expected to be positively related to negative emotions, but negatively related to wellbeing.

## Methods

### Participants and Procedures

Participants were 55 undergraduate students (43 women, 12 men, *M* age = 27.21 years, *SD* age = 6.81) recruited in a large French-Canadian university in Montreal. The sample size was based on recommended procedures of a minimum sample size of 5 five cases per free parameter in the model (Tabachnick and Fidell, 2001). Thus with 7 dependent variables, we needed at least 35 participants and we finally employed 55 participants. Participants received a $10 compensation for their participation. Upon their arrival, participants were informed of the general purpose of the study and gave their informed consent. Next, they were connected to physiological recording sensors and monitors measuring cardiac impedance (i.e., a measure of blood flow in the thoracic cavity; Frings et al., 2012), cardiac electrical activity using the electrocardiogram with a standard lead II configuration, respiration rate (RR) (i.e., mean respiration per minute) using a respiration belt transducer place around the chest, and blood pressure (BP) recorded by the CNAP™ monitor 500 (CNSystems Medizintechnik AG, Graz, Austria) using finger and arm cuffs. All physiological signals were acquired throughout the whole study using the Biopac MP160 physiological data acquisition system and the AcqKnowledge 5 software computed the physiological indexes. After the devices were in place, all participants were asked to complete a set of questionnaires and tasks in the following order: (a) engaging in a 5-min relaxation period (baseline), (b) demographic questions, (c) the Passion Scale, (d) the Challenge and Threat Appraisals Scale, (e) engaging in a timed education task, (f) the PANAS, (g) the Subjective Vitality Scale, and (h) engaging in a leisure task. Physiological reactions during the baseline and both tasks were used in our analyses (see below).

### Measures and Tasks

For all scales, except the PANAS, a 7-point scale was used (1 = *do not agree at all* to 7 = *very strongly agree*). For the PANAS, a 5-point scale (1 = *very slightly or not at all*, 5 = *extremely*) was used. For each scale, a mean score was calculated.

#### Relaxation Period

Based on previous research (e.g., Frings et al., 2012; Brimmell et al., 2019), participants engaged in a 5-min relaxation (baseline) period. Participants were asked to use relaxation methods they knew or to take deep breaths.

#### Demographic Questions

Participants’ age and gender were assessed.

#### Passion for University Studies

The Passion Scale (Vallerand et al., 2003; Marsh et al., 2013) consists of two 6-item subscales assessing OP (e.g., “I have difficulties controlling my urge to do my studies”; α = 0.69), and HP (e.g., “My studies are well integrated in my life”; α = 0.81).

#### Challenge and Threat Appraisals

The Cognitive Appraisals Scale (Skinner and Brewer, 2002; Berjot and Girault-Lidvan, 2009) includes two subscales measuring challenge appraisals (7-item subscale; e.g., “A challenging situation motivates me to increase my efforts,” α = 0.64), and threat appraisals (11-item subscale; e.g., “I lack self-confidence,” α = 0.94). Participants indicated how they feel “in general” because we wanted to examine the effects of cognitive appraisals on both the education and the leisure tasks.

#### Education Task

The education task was presented as an “excellent predictor of academic ability.” It consisted of five series of figures with one missing pattern (e.g., Raven’s matrices; Raven et al., 1998). Participants had to find which image correctly completed each series of illustrations in less than 45 s. The task was presented so as to induce stress in two ways. First, it was presented as a “predictor” of participants’ academic ability. Second, performance was timed to create a pressurized educational environment (for research on time constraints and stress, see Maule and Hockey, 1993) similar to a university exam setting. Participants were told that their performance would be calculated with reference to everyone else who has completed this task and that it would be based on the correctness of their answers and time taken to complete each matrix. Thus, it was impossible for them to guess if they had performed well or not on the task.

#### Positive and Negative Affect

The French version of the short Positive and Negative Affect Scale (PANAS; Watson et al., 1988; Gillet et al., 2013) includes two 5-item subscales assessing positive (e.g., “I feel interested”; α = 0.62) and negative emotions (e.g., “I feel upset”; α = 0.69). Participants indicated how they felt “at this very moment” following the education task.

#### Situational Subjective Vitality

A 5-item version of the Subjective Vitality Questionnaire (Ryan and Frederick, 1997) was used to assess participants’ situational subjective vitality after completing the education task (e.g., “I feel alive and vital.”; α = 0.89).

#### Leisure Task

The leisure task was clearly presented as an activity unrelated to participants’ studies. Participants were instructed that the task was similar to some they may engage in their “hobbies” and that “people do for fun.” Participants were presented with two words and they had to figure out which word was connected with both of them. Participants were asked to solve 10 such riddles.

#### Challenge / Threat Physiological Indices

Each physiological response such as heart rate, stroke volume (i.e., the volume of blood expelled by the heart per beat; Frings et al., 2012), and BP were scored as follows. For the baseline period (i.e., 5-min relaxation), only the data collected during the last 2 min were considered to calculate the mean scores of each physiological response (Frings et al., 2012). For the education and leisure tasks, the data collected during the whole task were considered to calculate the mean scores (Frings et al., 2012). A single physiological index of challenge and threat for each period (baseline, education task, and leisure task) was calculated using indices derived from the physiological measures. The heart rate and the stroke volume were multiplied to calculate the cardiac output (CO). The total peripheral resistance (TPR) was calculated using this formula: (mean BP / CO) × 80 (Sherwood et al., 1990). Then, the challenge / threat index was calculated as follows (see Blascovich et al., 2004; Brimmell et al., 2019). First, TPR and CO were converted in z scores. Then, TPR z scores were reverse scored before being summed with CO z scores. Higher scores on this index indicated challenge.

## Results

### Preliminary Analyses

There were no missing values in the data set. Box plots and Mahalanobis distances with a critical chi-square value at *p* = 0.001 revealed no univariate and multivariate outlier. Inspection of skewness indices showed that negative emotions were not distributed normally. Thus, we used a Log10 transformation to make data conformed more closely to the normal distribution (| skewness| < 1). Moreover, as shown by bivariate scatterplots and residual plots, all variables were related to each other in a linear manner. Variables also revealed no multicollinearity (VIF < 5). Means, standard deviations, and correlations between all variables are presented in Table 1. Moreover, to ensure that the education task created a pressurized environment as intended, we compared participants’ respiration rates (RR; number of respiration per minute) during the baseline period (i.e., relaxation), the education task, and the leisure task. A higher RR level indicates a higher activation level as would be found during higher stress levels. An analysis of variance (ANOVA) with repeated measures uncovered that participants’ RR was higher during the education task (*M* = 18.26) than during the baseline period (*M* = 15.41) and the leisure task (*M* = 17.67), Wilk’s Λ = 0.77, *F*_(2, 50)_ = 7.68, *p* = 0.001, ηp^2^ = 0.235. Similarly, participants’ heart rates during these three periods were compared. A higher HR level indicates higher engagement. An ANOVA with repeated measures unveiled that participants’ HR was higher during the education task (*M* = 79.53) than during the baseline period (*M* = 77.64) and the leisure task (*M* = 78.23), Wilk’s Λ = 0.78, *F*_(2, 52)_ = 7.56, *p* = 0.001, ηp^2^ = 0.23. There was no difference in RR and HR between the baseline period and the leisure task. Overall, these findings support the fact that participants experienced higher levels of stress and engagement during the education task than at other times during the session.

**TABLE 1:** Means, standard deviations, and correlations among the study variables.

	*M* (*SD*)	(1)	(2)	(3)	(4)	(5)	(6)	(7)	(8)	(9)
Harmonious passion (1)	5.18 (0.96)		0.12	0.29*	−0.04	0.32*	0.06	0.55*	−0.13	0.14
Obsessive passion (2)	3.04 (1.06)			0.08	0.35*	0.04	0.18	−0.03	−0.28^†^	0.20
Challenge appraisals (3)	5.02 (0.78)				−0.12	0.32*	0.03	0.43*	0.26^†^	0.28^†^
Threat appraisals (4)	3.94 (1.46)					−0.07	0.41*	−0.27*	0.05	0.13
Positive emotions (5)	3.13 (0.73)						−0.29*	0.63*	−0.24	−0.15
Negative emotions (6)	1.92 (0.74)							−0.15	0.20	0.35*
Subjective vitality (7)	4.48 (1.26)								0.04	0.12
Challenge/threat physiological index ET (8)	−0.10 (1.97)									0.70*
Challenge/threat physiological index LT (9)	−0.05 (1.91)									

*ET, education task; LT, leisure task.*

*p < 0.05.

*^†^p < 0.10.*

### Main Analyses

The proposed model posited that HP for one’s studies should be positively related to challenge appraisals and OP should be positively related to threat appraisals. In turn, the challenge cognitive appraisals should be positively related to the challenge/threat physiological indices, positive emotions, and situational subjective vitality. On the other hand, the threat cognitive appraisals should be positively related to negative emotions and negatively to situational subjective vitality. The model was composed of two exogenous variables (i.e., HP and OP) and seven endogenous variables (i.e., challenge and threat cognitive appraisals, challenge/threat physiological indices during the education and leisure tasks, positive and negative emotions, and situational subjective vitality). To test the hypothesized model, a path analysis was conducted using MPlus version 8.6 (Muthén and Muthén, 1998–2017). Paths from HP to challenge appraisals and from OP to threat appraisals were specified, followed by paths from challenge appraisals to the challenge / threat physiological indices during the education and leisure tasks, positive emotions, and subjective vitality. In addition, paths were drawn from threat appraisals to negative emotions and subjective vitality. Finally, covariances among the two exogenous variables and among the error terms were estimated.

**FIGURE 1:**
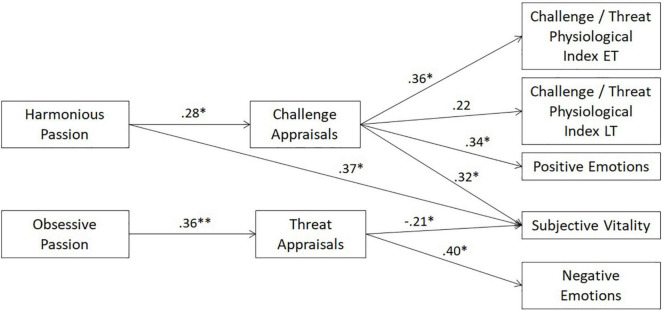
Results of the Path Analysis. Standardized path coefficients are presented. For clarity concerns, covariances are not presented. *N* = 55. ET, education task; LT, leisure task. *p < 0.05 and ^**^p < 0.01.

Results indicated that the hypothesized model did not have an acceptable fit to the data. In line with recommended procedures (Kline, 2015), visual inspection were conducted and suggested adding a positive path from HP to subjective vitality. Such a path is in line with previous findings (e.g., Vallerand et al., 2007, 2008; Dubreuil et al., 2014). This modified model had good fit to the data (see Kline, 2015), χ^2^ = 16.70, *df* = 15, *p* = 0.337; RMSEA = 0.05 [0.00, 0.14], *p* = 0.476; CFI = 0.99; TLI = 0.97; SRMR = 0.06. The standardized solutions are presented in Figure 1. Results showed that HP was positively associated with challenge appraisals (β = 0.28, *p* = 0.011) whereas OP was positively related to threat appraisals (β = 0.36, *p* = 0.002). In turn, the challenge appraisals were positively related to positive emotions (β = 0.34, *p* = 0.001), subjective vitality (β = 0.32, *p* = 0.002), the challenge / threat physiological index during the education task (β = 0.36, *p* = 0.026), but not significantly with the challenge / threat physiological index during the leisure task (β = 0.22, *p* = 0.162). By contrast, the threat appraisals were positively associated with negative emotions (β = 0.40, *p* = 0.001) and negatively related to subjective vitality (β = −0.21, *p* = 0.012). Finally, there was a positive path from HP to subjective vitality (β = 0.37, *p* = 0.001).

Indirect effects (see Table 2) were significant or approaching significance, except in one case. All bias-corrected 95% bootstrapped confidence intervals, except one, did not include zero, which supports the meaningful effects of challenge and threat appraisals as mediators of the relationships between passion and the outcome variables.

**TABLE 2:** Bootstrap estimates of the indirect effects and their associated bias-corrected 95% confidence intervals.

Predictor	Mediator	Outcome	β	95% CI	*p*-values
HP	Challenge appraisals	Challenge/threat physiological index ET	0.10	[0.01, 0.28]	*p* = 0.065
HP	Challenge appraisals	Challenge/threat physiological index LT	0.06	[−0.01, 0.19]	*p* = 0.186
HP	Challenge appraisals	Positive emotions	0.10	[0.02, 0.24]	*p* = 0.057
HP	Challenge appraisals	Subjective vitality	0.09	[0.02, 0.22]	*p* = 0.056
OP	Threat appraisals	Subjective vitality	−0.08	[−0.17, −0.02]	*p* = 0.058
OP	Threat appraisals	Negative emotions	0.14	[0.04, 0.32]	*p* = 0.035

*HP, harmonious passion; OP, obsessive passion; ET, education task; LT, leisure task.*

In sum, the present findings confirmed the differential role of HP and OP in the way individuals appraised stressful situations related to their passion. Because HP led to challenge appraisals, it was associated with adaptive outcomes both at the psychological and physiological levels. On the other hand, through threat appraisals, OP led to less adaptive outcomes. These findings replicate past research on the role of passion in outcomes and extend the processes outlined by Lavoie et al. (2021) at the physiological level with cardiovascular responses.

## Discussion

Overall, the results of the present study lead to at least three major conclusions. First, these findings replicated past research on the role of HP and OP in leading, respectively, to positive and negative emotions (see Curran et al., 2015; Vallerand, 2015 for reviews). They also replicated the mediating role of challenge and threat appraisals, respectively, in the HP-positive emotions and OP-negative emotions relationships (Lavoie et al., 2021). Thus, the type of passion (HP or OP) does matter as pertains to emotions and determines the outlook that one holds in the situation (challenge vs. threat) and the type of emotions that ensue. Of additional interest, this analysis also applies to a measure of wellbeing, namely situational vitality. HP positively predicted situational vitality, both directly and indirectly through challenge appraisals, while OP negatively predicted situational vitality through threat appraisals.

A second conclusion is that cognitive appraisals were found to also predict cardiovascular reactivity. Specifically, challenge appraisals led to adaptive cardiovascular indices whereas threat appraisals were not related to such physiological measures. In addition, the present findings suggest that HP creates the onset of adaptive psychological and physiological responses whereas the responses are less adaptive with OP. Furthermore, as hypothesized such effects seem to be stronger on the task related to participants’ passion for academia (education task) than on the other task (leisure task). These are the *first* findings to show that passion (and especially HP) can influence physiological measures through its relationship with cognitive appraisals. Future research is needed to clarify the role of OP in physiological responses. Future research is also necessary to see if the physiological effects found in this study can be replicated in other achievement-oriented life domains such as work, sports, and music and the arts.

A final conclusion pertains to the field of positive psychology. Most studies in the field have relied on self-report measures (see Kim et al., 2018). Although such scales are useful, especially when valid and reliable, it nevertheless remains that the use of objective indicators is important to solidify a field that has largely relied on self-report measures. Some research in positive psychology has recently started to use physiological measures (e.g., Cousin et al., 2020). However, the present research is the first to show correspondence between passion and cardiovascular reactivity measures, thereby extending the field of passion and positive psychology in new and exciting directions. For instance, research has shown that using one’s strengths at work leads to HP for work that in turn facilitates increased performance and psychological wellbeing (Dubreuil et al., 2014). Is it possible that using one’s strengths opens up a path of challenge rather than threat, thereby facilitating adaptive cardiovascular responses as well? Future research along this path is clearly encouraged.

This research has some limitations. First, the sample size was limited and no control variables were used. Therefore, future research should be conducted with a higher number of participants and with covariates to identify more precisely the role of passion and cognitive appraisals in participants’ psychological and physiological responses. Secondly, although the physiological measures suggested that the education task was more stressful than the leisure task, future research should counterbalance the two tasks. Finally, a different cognitive appraisals scale should be used in future research. Indeed, the cognitive appraisals scale used in this research has theoretical roots that diverge from the work of Lazarus (1991, 1999). As posited by Meijen et al. (2020), a cognitive appraisals scale could be developed in future research to better reflect the components of the Lazarus’ model.

In sum, the present findings suggest that HP creates the onset of adaptive challenge-oriented psychological and physiological responses, which may indicate some resilience as shown by a positive adaptation following a stressful situation (Fletcher and Sarkar, 2013). On the other hand, OP is conducive to threat-oriented and less adaptive responses. Future research along those lines, where the psychological and physiological dimensions are weaved in together, should extend the field of positive psychology in exciting new directions.

## Data Availability Statement

The raw data supporting the conclusions of this article will be made available by the authors, without undue reservation.

## Ethics Statement

The studies involving human participants were reviewed and approved by the Comité Institutionnel d’Éthique de la Recherche Avec des Êtres Humains de l’UQAM. The patients/participants provided their written informed consent to participate in this study.

## Author Contributions

RV wrote the Introduction and Discussion sections and worked on the conceptualization of the questionnaire and the final model. VP wrote parts of the Introduction and Discussion sections, also wrote the Methods and Results sections, collected some of the data, analyzed the data, and worked on the conceptualization of the questionnaire and the final model. CR collected and cleaned the data, worked on the conceptualization of the questionnaire, created the research protocol, and set up the physiological measures. All authors contributed to the article and approved the submitted version.

## Conflict of Interest

The authors declare that the research was conducted in the absence of any commercial or financial relationships that could be construed as a potential conflict of interest.

## Publisher’s Note

All claims expressed in this article are solely those of the authors and do not necessarily represent those of their affiliated organizations, or those of the publisher, the editors and the reviewers. Any product that may be evaluated in this article, or claim that may be made by its manufacturer, is not guaranteed or endorsed by the publisher.
